# Vitamin C–Based Gingival Depigmentation Versus Surgical Depigmentation: A Randomized Clinical Trial

**DOI:** 10.1155/tswj/3299188

**Published:** 2024-12-31

**Authors:** Aehad Ul Haque, Nand Lal, Shalini Kaushal, Pavitra Rastogi, Rameshwari Singhal

**Affiliations:** Department of Periodontology, Faculty of Dental Sciences, King George's Medical University, Lucknow, Uttar Pradesh, India

**Keywords:** depigmentation, gingiva, vitamin C

## Abstract

**Background:** This study aimed at comparing gingival depigmentation by locally injected vitamin C with surgical depigmentation, in terms of effectiveness and patient acceptability.

**Methods:** Forty-two patients presenting with ethnicity-related hyperpigmentation were randomly divided into two groups, Group I (*n* = 21) was treated with locally injected vitamin C and Group II (*n* = 21) was treated by surgical depigmentation. The outcome was assessed using Gingival Pigmentation Index (GPI) and Skin Hyperpigmentation Index (SHI).

**Results:** Both the techniques were successful in treating gingival hyperpigmentation, but the patients who underwent vitamin C–based depigmentation showed significantly higher satisfaction with the treatment result, based on patient reported Visual Analog Scale (VAS) scoring.

**Conclusion:** Based on the findings of this study, it was concluded that locally injected vitamin C and surgical depigmentation are comparable in their effectiveness for treating gingival hyperpigmentation.

**Trial Registration:** ClinicalTrials.gov identifier: CTRI/2023/02/050127.

## 1. Introduction

The white esthetics of a tooth and pink esthetics of gingiva are two building blocks of a harmonious smile [1, 2]. Gingival color is governed by various factors such as, ethnicity, pregnancy, smoking, and drug intake [3–5]. Its histologic determinants include vasculature, keratinization, and pigments such as melanin [6, 7]. Melanin is a brown colored, non–hemoglobin-derived pigment produced by melanocytes found in gingival epithelium [8, 9]. They bear an enzyme called tyrosinase which catalyzes the hydroxylation of L-tyrosine to L-Dopa (levo-dihydroxyphenylalanine), which then gets converted to dopaquinone [10]. Upon further conversion, dopaquinone either forms the brownish black eumelanin or the reddish-yellow pheomelanin [11]. Eumelanin is generally more abundant in human gingiva [12].

Melanin pigmentation of gingiva is a normal genetic trait, but many affected individuals still find it unesthetic [13]. A gingival color darker beyond what is normally expected is referred to as gingival hyperpigmentation, and its reduction is termed as gingival depigmentation [14, 15]. The modalities of depigmentation range from simple techniques, such as surgical depigmentation, to more advance methods, such as laser ablation and cryotherapy [16–19].

The surgical technique is widely considered a gold standard for gingival depigmentation, but like most other depigmentation modalities, it is resective in nature, as it involves removal of some amount of gingival tissue [20]. More recently, nonresective methods such as nonablative laser depigmentation, transepithelial depigmentation, and vitamin C–based mesotherapy have gained popularity [21–23]. The antimelanogenic effect of vitamin C on gingival pigmentation was first demonstrated in 1961 [24]. A recent study has showed that it inhibits tyrosinase activity in melanocytes by intracellular acidification [25]. Since tyrosinase is essential for the conversion of L-tyrosine to L-dopa (a rate limiting step), its inhibition results in downregulation of melaninogenesis [26].

The present study sought to evaluate the effectiveness of locally injected vitamin C in treating physiologic gingival hyperpigmentation in human subjects. The technique was also compared with surgical depigmentation in terms of efficacy and patient satisfaction with the treatment result.

## 2. Materials and Methods

### 2.1. Methodology

This single-center study was conducted at periodontology out-patient department, in accordance with the principles of the Declaration of Helsinki, 2002, and its amendments [27]. It was a randomized parallel-group active controlled trial with an allocation ratio of 1:1. Prior to study commencement, ethical clearance was obtained from the Institutional Ethics Committee (Ref. Code: XI-PGTSC-IIA/P12). A written informed consent was obtained from each study participant prior to enrollment.

### 2.2. Study Population

The determination of sample size was done using a previous study [28]. Sample size at 90% power was calculated within the follow-up time in two groups using the following formula on the basis of variation in gingival pigmentation index (GPI) [29].

(1) $$\begin{array}{c} n=k\frac{{\left(z_{\alpha }+z_{\beta }\right)}^{2}\left(\sigma_{1}^{2}+\sigma_{2}^{2}\right)}{d^{2}}, \end{array}$$

where *σ*1 = 1.04, the SD of the pigmentation index in the first group, *σ*2 = 1.377, the SD of the pigmentation index in the second group, *d* = max (*σ*1 and *σ*1), the minimum difference considered clinically significant, *k* = 1.0, the design effect, Type I error *α* = 5% corresponding to 95% confidence level, Type II error, and *β* = 10% for detecting results with 90% power of study.

So, the required sample size for each group was 21.

**Figure 1:**
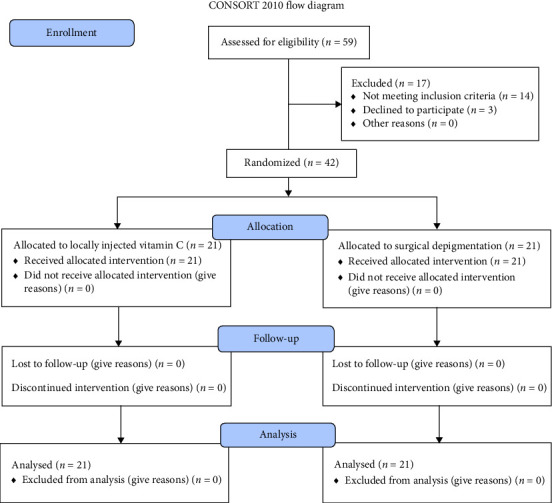
CONSORT flow diagram.

A total of 59 patients were assessed for eligibility, and 17 patients were excluded for not meeting the eligibility criteria (Figure 1). A total of 42 patients were included in the study and randomly divided into two groups of 21 each.

The patients were informed about the aims, objectives, and purpose of the study, and those willing to participate were assessed as per the following criteria:

#### 2.2.1. Inclusion Criteria

The inclusion criteria include the following:

1. Patients ≥ 18 years of age
2. Patients with hyperpigmentation in the anterior gingiva
3. Patients willing to get treated for gingival hyperpigmentation
4. Systemically healthy patients

#### 2.2.2. Exclusion Criteria

The exclusion criteria include the following:

1. Patients with a history of smoking or tobacco intake
2. Pregnant or lactating women
3. Patients exhibiting 1.5 mm or less gingival thickness in the region of anterior gingiva (thickness was measured under local anesthesia, by inserting an endodontic spreader perpendicular to the gingival surface, in the attached gingiva of each tooth, and repositioning its stopper, followed by measurement of the distance between the stopper and the tip of the spreader using a digital caliper)
4. Patients who consumed drugs that may affect gingival pigmentation
5. Patients with systemic diseases which could cause gingival hyperpigmentation
6. Patients with oral or periodontal diseases which could affect the gingival color

### 2.3. Randomization

The included subjects were sequentially numbered as per the date of enrollment, and randomization was done using the National Cancer Institute's Randomization tool (https://ctrandomization.cancer.gov/), which is based on the asymptotic maximal procedure [30]. Patient enrollment and allocation were overseen by coinvestigators and revealed to principal investigator only after randomization. Statistical analysis was overseen by a coinvestigator who was blinded to intervention allocation. The patients were divided into two groups, and a single calibrated operator (principal investigator) performed the depigmentation procedures as follows:

• Group I: Gingival depigmentation using locally injected vitamin C
• Group II: Gingival depigmentation using surgical blade

### 2.4. Gingival Depigmentation by Locally Injected Vitamin C

**Figure 2:**
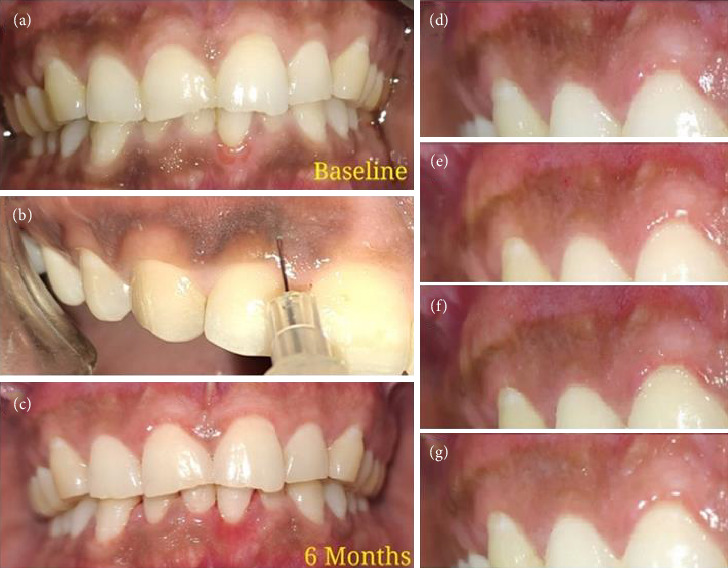
(a) Clinical picture before treatment, (b) gingival depigmentation by locally injected vitamin C, (c) clinical picture after treatment, (d) 1 week after first session, (e) 1 week after second session, (f) 1 week after third session, and (g) 1 week after fourth session.

Firstly, topical anesthesia was administered in the gingiva corresponding to anterior teeth using a sprayable solution of 15% lignocaine (one spray per quadrant). The area was then dried, and 1.25 mL of 10% vitamin C solution was injected into each quadrant of the anterior gingiva through multiple pricks, using an insulin syringe with a 30-gauge needle (Figure 2). The needle was inserted parallel to the gingival surface, and about 0.1 mL solution was introduced at each point of injection. After each session, the patients were prescribed with oral analgesics (aceclofenac 15 mg + paracetamol 325 mg) to be taken twice daily for the first 24 h after the procedure and as per need thereafter. The same procedure was repeated at weekly intervals up to a maximum of four visits or till no further color change could be observed, whichever occurred earlier.

### 2.5. Gingival Depigmentation Using Surgical Blade

**Figure 3:**
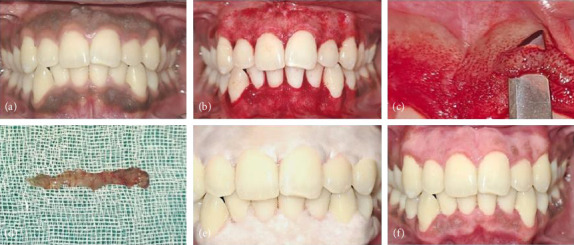
(a) Clinical picture before treatment, (b) surgical depigmentation of gingiva, (c) removal of hyperpigmented tissue, (d) removed hyperpigmented tissue, (e) periodontal dressing, and (f) clinical picture after treatment.

Local anesthesia was attained by infraorbital and mental nerve blocks using an injectable anesthetic solution of 2% lignocaine with 1:80000 adrenaline. Upon confirmation of adequate anesthesia, the pigmented gingival tissue was carefully excised using a 15-number surgical blade, without exposing the underlying bone (Figure 3). Needful gingivoplasty was then performed, and a periodontal dressing was placed on the surgical site for 1 week (COE-PAK, GC America Inc.). The patient was prescribed with analgesics (aceclofenac 15 mg + paracetamol 325 mg to be taken twice daily on the first day after procedure and as per need thereafter).

#### 2.5.1. Study Variables

The following variables were recorded for each patient by the principal investigator:

1. GPI [30].
It was assessed at baseline, 1 month follow-up and 6-month follow-up. Patient's gingiva was visually inspected and one of the following scores was assigned:
  0: Absence of pigmentation
  1: Spots of brown to black color or pigments
  2: Brown to black patches but no diffuse pigmentation
  3: Diffuse brown to black pigmentation in marginal and attached gingiva
2. Skin Hyperpigmentation Index (SHI) [31].
SHI is a computer-based index which uses brown pixel histogram profiling of clinical images to derive “Pigmentation Scores” (PS) for hyperpigmented and normal tissues, which are then used to derive the “SHI” as follows:
  (2) $$\begin{array}{c} \text{SHI score}=\frac{\text{PS of Hyperpigmented Area}}{\text{PS of Normal Reference Point}}. \end{array}$$
The score ranges from 1 to 4, where 1 denotes no difference and 4 denotes maximum hyperpigmentation. In the current study, the darkest region of anterior gingiva was taken as the reference point for hyperpigmentation, and the skin of the tip of the nose was taken as the reference point for the normal skin. The clinical photographs for SHI estimation were taken by the same operator under similar ambient light conditions, and the scores were recorded for each patient at baseline, 1-month postoperatively, and 6-month postoperatively.
3. Patient satisfaction analysis using Visual Analog Scale (VAS) [32].
It was carried out at 6-month follow-up, using a 10-point graded VAS, provided as a printed 10 cm long line with 10 divisions, numbered from 0 to 10. The patients were instructed to make a handwritten mark on the line to denote their satisfaction. Each centimeter on the line measured from the extreme left denoted 10% satisfaction. For example, a mark made by the patient, 2 cm away from the extreme left, was inferred as 20% satisfaction.

### 2.6. Statistical Analysis

The results were analyzed using descriptive statistics and by making comparisons among study groups. Quantitative (discrete) data were summarized as mean ± SD. The comparison of the SHI was done using unpaired *t*-test as the scores showed a normal distribution on Q-Q plot and Shapiro–Wilk test. The datasets for GPI and VAS scores did not show a normal distribution, so they were assessed using the Mann–Whitney test. The Mann–Whitney *U* test is used to compare differences between two independent groups when the dependent variable is either ordinal or continuous, but not normally distributed.

## 3. Result

**Figure 4:**
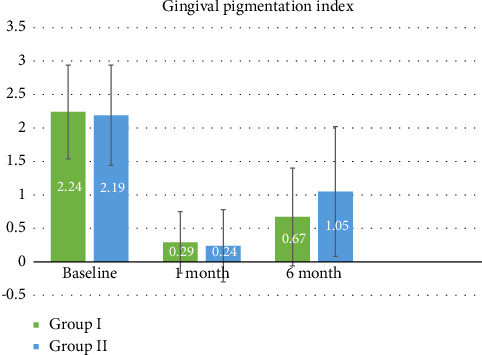
Graph of Gingival Pigmentation Index (GPI) change.

**Figure 5:**
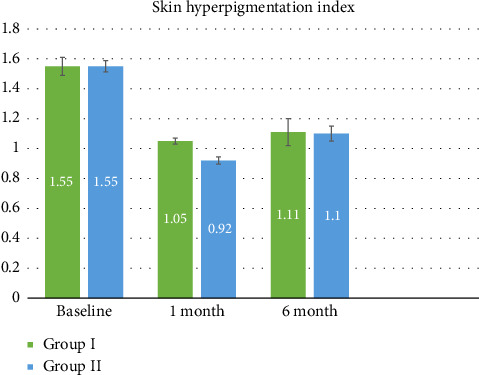
Graph of Skin Hyperpigmentation Index (SHI) change.

**Figure 6:**
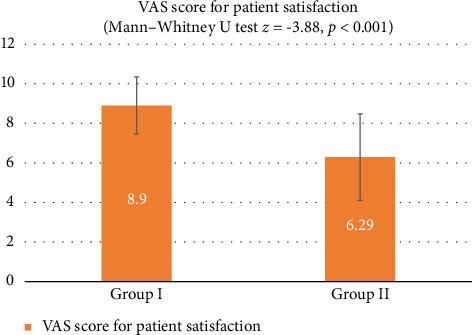
Graph of Visual Analog Scale (VAS) scores given by the patients.

A total of 42 patients were enrolled and randomly divided into two groups. Group I (*n* = 21) was treated using depigmentation by locally injected vitamin C, and Group II was treated by surgical depigmentation. Each patient was followed-up for 6 months, and no dropouts were seen. The participant characteristics have been summarized in Table 1, and statistical analyses of GPI, SHI, and VAS have been graphically represented in Figures 4, 5, and 6, respectively. At baseline, no statistically significant intergroup difference was noted in the GPI scores (Mann–Whitney test, *p* = 0.859). At the 1-month postoperative follow-up, the mean scores got reduced in both the groups, but no significant intergroup difference could be observed, (Mann–Whitney test, *p* = 0.541). At 6 months, the mean GPI scores got slightly raised in each group, but they were still comparable (Mann–Whitney test, *p* = 0.211).

The baseline SHI scores showed no significant intergroup difference (unpaired *t*-test, *p* = 0.962), but a significant difference was noted at 1 month follow-up (unpaired *t*-test, *p* < 0.001), with Group II showing greater reduction in SHI scores. However, at 6-month follow-up, there was no significant intergroup difference in the SHI (unpaired *t*-test, *p* value = 0.891). The mean VAS score for Group I was 8.90 (SD = 1.45), while for Group II, it was 6.29 (SD = 2.19). Group I showed a significantly higher satisfaction according to the VAS (Mann–Whitney test, *p* value < 0.001), indicating a 26.1% higher patient satisfaction in vitamin C group patients.

## 4. Discussion

The present study compared gingival depigmentation by locally injected vitamin C (Group I), with conventional surgical depigmentation (Group II) in patients of ethnicity-related gingival pigmentation. Four sessions of vitamin C administration were carried out at weekly intervals for Group I patients, such that a total of 5 mL ascorbic acid was administered in each session. A similar regimen has previously been used in another clinical study [28]. The effect of treatment was assessed using GPI but since it is solely based on clinical observation, it lacks reliability. So, for a more objective assessment, computer-based SHI was used. The SHI has already been used in a few studies which assessed the skin, but to the best of authors' knowledge, this is the first study to use this index for analyzing gingiva [33, 34].

The improvement in the gingival color was initially more marked in the surgical depigmentation group, while vitamin C group patients exhibited a gradual lightening of gingiva, as follows:

1. Immediately upon injection: Gingiva got temporarily blanched
2. 1 week after first session: Areas of least pigmentation turned pink, and darker areas began to get interspersed with pink dots
3. 1 week after second session: Pink areas started to expand
4. 1 week after third session: Hyperpigmented areas became faint
5. 1 week after fourth session: Almost pink gingiva could be noted

The gradual improvement in gingival pigmentation with multiple sessions of vitamin C injection has also been reported by other studies [35, 36].

The intergroup comparison of the GPI showed no statistically significant difference at 1-month and 6-month postoperative follow-ups, suggesting that effectiveness of both the techniques is comparable. These findings corroborate with the observations of a recent study which found no significant difference between the effectiveness of vitamin C–based depigmentation and surgical depigmentation [37]. The intergroup comparison of SHI scores at 1-month and 6-month follow-ups indicated that surgical depigmentation had significantly better initial results at 1 month follow-up. The basis for this trend may be the fact that gingiva, which undergoes surgical depigmentation, heals by secondary intention, and the newly formed epithelium is initially devoid of melanin. This observation has also been reported in a previous study which found that surgical depigmentation shows better results initially, but the effect diminishes thereafter [28]. On the other hand, vitamin C depigmentation is based on the inhibition of new melanin formation, so the color change becomes evident more gradually as the gingival turnover occurs. Nevertheless, the results become comparable over 6 months because repigmentation occurs, as neither of the techniques can permanently eliminate the melanin-forming ability of gingival epithelium.

After treatment completion, each subject underwent a VAS-based patient satisfaction analysis. Mean VAS scores for Group I and Group II were 8.90 and 6.29, respectively. The vitamin C group patients exhibited a significantly higher satisfaction with the treatment result. This is likely because of the minimal invasiveness of this technique that patient compliance with vitamin C–based depigmentation is better. A previous study which compared patient satisfaction in patients of vitamin C–based depigmentation with patients who underwent laser depigmentation also found comparable results [38]. However, VAS scoring is prone to subjectivity due to the possible variations in individual patient preferences, so more comprehensive methods of the patient satisfaction analysis must be employed in future research.

No major postoperative complications were reported by any patients of the surgical depigmentation group, and only minimal discomfort was experienced by three patients postoperatively. A similar observation had also been reported by other authors [39]. Two patients of the vitamin C group experienced slight pain in the treated area immediately after each session. A similar postoperative observation has also been reported in a recent clinical study [40]. Findings from the present study suggest that vitamin C–based gingival depigmentation is efficacious in treating gingival hyperpigmentation. Similar conclusions have also been drawn in other recent studies, but the technique for gingival depigmentation must carefully be chosen by the clinician based on individual patient characteristics such as gingival phenotype, heredity, and esthetic demands of the patient [41]. In addition, since vitamin C–based depigmentation requires multiple visits, patient compliance must also be considered prior to treatment selection.

Although this study provided an interesting insight into the understanding of vitamin C–based gingival depigmentation, it had a short follow-up. Another major limitation of this study was that no index was included in the study parameters for assessing gingival phenotype, although it may have a significant effect on the treatment outcome [28]. In addition, only the gingiva corresponding to the anterior teeth was included, while patients with premolar to premolar or molar to molar smile may have greater gingival display and may require additional treatment. The treatment procedures and recording of clinical parameters were carried out by the same investigator (principal investigator), which may have led to bias. Future investigations with larger sample size and longer follow-ups are warranted to confirm the findings of this study.

## 5. Conclusion

This study concludes that gingival depigmentation by locally injected vitamin C was as efficacious as surgical depigmentation of gingiva. It also concluded that vitamin C–based gingival depigmentation renders a greater patient satisfaction with the treatment result, as compared to surgical depigmentation of gingiva.

## Figures and Tables

**Table 1:** Baseline data.

Baseline data	Group I (depigmentation by locally injected vitamin C)		Group II (surgical depigmentation)		Test of significance for intergroup comparison	*T*-value/chi-square value for intergroup comparison	*p* value for intergroup comparison
	Mean or %	Standard deviation	Mean or %	Standard deviation			
Age (years)	29.86	9.50	24.52	11.25	Unpaired *t* test	1.66	0.105
Males	38.1%		61.9%		Chi-square test	2.38	0.123
Females	61.9%		38.1%				

## Data Availability

The data that support the findings of this study are available from the corresponding author upon reasonable request.

## Supporting Information

1. CONSORT checklist.
